# Acupuncture Differentially Affects the High-Frequency Spectral Energy in Radial Pulse Positions Depending on Type of Lower Back Pain

**DOI:** 10.1155/2019/4024501

**Published:** 2019-11-21

**Authors:** Hui-Ping Ng, Chin-Ming Huang, Wen-Chao Ho, Yu-Chen Lee

**Affiliations:** ^1^International Master Program in Acupuncture, China Medical University, Taichung City 40402, Taiwan; ^2^Singapore Chung Hwa Medical Institution, Singapore; ^3^School of Post-Baccalaureate Chinese Medicine, China Medical University, Taichung City 40402, Taiwan; ^4^Department of Public Health, China Medical University, Taichung City 40402, Taiwan; ^5^Department of Nursing, Asia University, Taichung City 41354, Taiwan; ^6^Department of Acupuncture, China Medical University Hospital, Taichung City 40402, Taiwan; ^7^Graduate Institute of Acupuncture Science, China Medical University, Taichung City 40402, Taiwan; ^8^Chinese Medicine Research Center, China Medical University, Taichung City 40402, Taiwan

## Abstract

Acupuncture is a core discipline in traditional Chinese medicine (TCM) and has been practised in China for centuries. In traditional acupuncture, pulse palpation is an important clinical diagnostic technique that guides practitioners in their treatment strategies as they evaluate the effectiveness of the treatment. This paper provides the findings of our investigation of acupuncture's effect on specific radial pulse spectral energies in 41 individuals with lower back pain (LBP), in response to a single acupuncture treatment delivered bilaterally at acupoints BL23, BL25, and BL40. Baseline assessments (vital signs and radial pulse diagnoses), primary outcome measures (radial pulse diagnoses), and secondary outcome measures (the Faces Pain Scale-Revised (FPS-R) and fingertip-to-floor (FTF) tests) were performed at specified intervals before and after the intervention. Our study provides novel information about the effects of acupuncture on the radial pulse spectral energy in individuals with different types of LBP. Our findings suggest that the right *Chi* pulse is an effective indicator to assess the effects of acupuncture in individuals with fixed, distended, or sharp pain, whereas the left *Guan* pulse is a potentially useful diagnostic technique to determine acupuncture's effects in individuals with dull, aching pain. The acupoints BL23, BL25, and BL40 provide effective treatment for LBP. Study participants with dull, aching pain had a significant improvement in their lumbar ranges of motion, and their pain rating scores were markedly decreased after acupuncture treatment.

## 1. Introduction

Acupuncture is a core discipline in traditional Chinese medicine (TCM) and has been practised in China for centuries. In traditional acupuncture, pulse palpation is an important clinical diagnostic technique that guides acupuncture practitioners in their treatment strategy and evaluates the effectiveness of the treatment [1]. The *Ling Shu* text in Chapter 1 of *Huang Di Nei Jing* advises that “one must first diagnose the pulse before acupuncture, treat the disease only after perceiving the severity or ease of the *Qi*” [2].

Conventionally, TCM practitioners palpate the radial pulse with the second, third, and fourth fingers individually or simultaneously, at the three regions of each wrist. These regions, representing the *Cun* (distal), *Guan* (middle), and *Chi* (proximal) pulses (the *Pin Yin* pulses named *Cun*, *Guan*, and *Chi* are adopted in this article; they are also translated as *Chun*, *Guan*, and *Chi* pulses in some references), correspond to the respective visceral organs in TCM: right *Cun* (lung and chest), right *Guan* (stomach and spleen), right *Chi* (kidney and lower abdomen), left *Cun* (heart), left *Guan* (liver and gallbladder), and left *Chi* (kidney and lower abdomen) [3].

Each radial pulse profile has unique characteristics: depth, rate, waveform, density, and intensity. The ancient texts describe 28 common pulse profiles observed in clinical practice [4]. These are categorised by (1) depth: floating, sunken, C*hi* (proximal), or *Cun* (distal); (2) rate: slow, rapid, surging, or intermittent; (3) waveform: long, short, narrow, broad, thick, thin, rough, fine, firm, or gentle; and (4) intensity and density: converging, dispersing, extending, shortening, moving forward, moving backward, moving upward, or moving downward [3]. Each pulse profile provides information about the physiological conditions or disorders in the human body [5]. For example, Jeon et al. studied pulse wave variation during the menstrual cycle and reported that the pulse appeared to be either string-like or slippery and rapid [6].

The accuracy of pulse diagnoses depends on the finger sensitivity of TCM practitioners and their experiential perception. Inconsistencies in diagnoses and treatments can result from subjective assessments by TCM practitioners [7, 8]. Since the 1950s, quantitative analyses of pulse profiles have been studied by researchers [1]. The development of the pulse sphygmograph, providing quantitative and graphical presentations of the radial pulse, is an important milestone in transforming conventional pulse diagnoses into scientific assessments.

This paper provides the findings of our quasi-experimental investigation on the effects of acupuncture on the specific radial pulse spectral energy (SE) in participants with lower back pain (LBP). Using the pulse sphygmograph, we investigated high-frequency SE (SE_13–50 Hz_) in the radial pulse during the pre- and postintervention stages. An objective assessment of the ranges of motion using the fingertip-to-floor (FTF) test and a subjective assessment of the Faces Pain Scale-Revised (FPS-R) test were included to determine the effectiveness of acupuncture in different types of LBP. In addition, the Health Status Questionnaire, the Oswestry Disability Index (ODI), and the Constitution in Chinese Medicine Questionnaire (CCMQ) were used to determine the overall health status of each study participant. Full details of the methodologies are described in the study protocol “An investigation into the effects of acupuncture on radial pressure pulse waves in patients with low back pain: a protocol for a quasi-experimental study” [1].

## 2. Materials and Methods

### 2.1. Study Setting and Design

**Figure 1:**
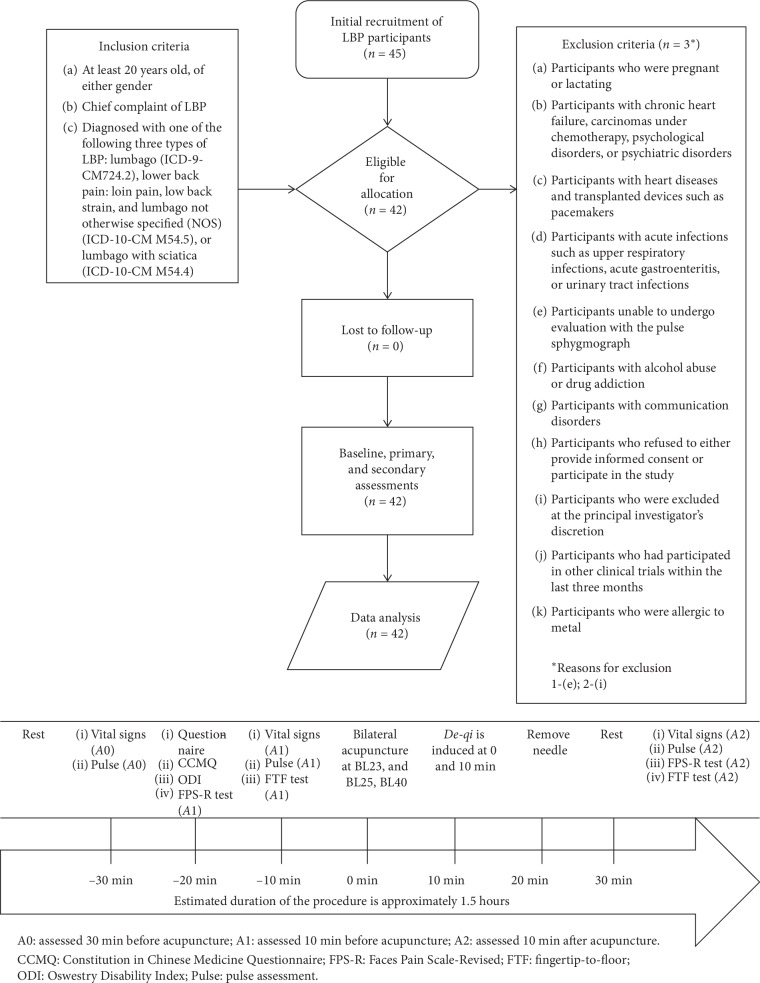
The study flow diagram and study procedure. All enrolled patients underwent a baseline assessment of the radial pulse spectral energy (SE) and the vital signs and the Faces Pain Scale-Revised (FPS-R) test. The study applied the Health Status Questionnaire, the Oswestry Disability Index (ODI), and the Constitution in Chinese Medicine Questionnaire (CCMQ). The SE, vital signs, and fingertip-to-floor (FTF) test were assessed during the pre- and postinterventions.

The study was conducted in the Acupuncture Department of the China Medical University Hospital (CMUH), Taichung, Taiwan, from April 2018 through March 2019. The Standard Protocol Items: Recommendations for Interventional Trials (SPIRIT) 2013 checklist was used to guide this single-arm, nonrandomised, quasi-experimental study. We adopted the Transparent Reporting of Evaluations with Nonrandomised Designs (TREND) guideline to report the findings.  Figure 1 provides details of the study flow diagram and study procedure.

### 2.2. Study Participants

Recruitment into the study was advertised on the hospital's regular internal circulars, bulletin boards, and websites. During the recruitment phase, 42 eligible individuals aged at least 20 years with a primary complaint of LBP, based on the predefined inclusion and exclusion criteria, were invited to participate in the study by the chief attending physician. Informed consent including the study aims, description of the procedures, and potential risks was approved by the CMUH Research Ethics Committee (REC) and was communicated to each individual, who provided written informed consent before the procedure.

### 2.3. Interventions

Each participant received a single acupuncture treatment in this research study. During the procedure, the participant rested in a prone position while acupuncture needles were inserted bilaterally at acupoints BL23 (Shenshu), BL25 (Dachangshu), and BL40 (Weizhong) as defined in *WHO Standard Acupuncture Point Locations in the Western Pacific Region* [9]. These locations, at the Bladder meridian of the lower back region and the transverse crease of the popliteal fossa, are common proximal and distal acupoints used in the treatment of LBP. The *Ling Shu* text in Chapter 51 of *Huang Di Nei Jing* states that the Shenshu (BL23) acupoint is the Back-Shu point of the kidneys, with functions including tonifying the kidneys and fortifying Yang, nourishing kidney *Yin*, firming kidney *Qi*, and strengthening the lumbar regions. The Dachangshu (BL25) acupoint is the Back-Shu point of the large intestine, which strengthens the lumbar regions and legs. The Weizhong (BL40) acupoint is one of the four command acupoints, which indicates “look for the Weizhong (BL40) acupoint to relieve disorders of the waist and back” [10].

The acupuncture needles were inserted within the defined safe range of 12.5–25 mm for BL25 and BL40, and within 12.5–40 mm for BL23 [11]. The needles were retained for 20 min after the needling sensation (*De-qi*) was manually stimulated with sufficient stimulus intensity during insertion and subsequently repeated 10 min after the insertion. A similar stimulus intensity was applied by the same acupuncturist to all study participants without supplementing or draining techniques [12]. In acupuncture practice, supplementing and draining techniques are generally applied to increase *Qi* in the deficiency state and decrease *Qi* in the repletion state after the patient experiences the *De-qi* sensation [13]. In our study, we avoided using these techniques, which may have resulted in confounding bias in the results.

### 2.4. Objectives and Hypothesis

Controversial explanations of the relationship between bodily organs and *Chi* pulse positions are documented in various ancient texts. For instance, the *Suwen* text in Chapter 17 of *Huang Di Nei Jing* states that “both *Chi* pulse positions correspond with the kidney and abdomen” [3, 14]; *Nan Jing*: *The Classic of Difficult Issues* documents that the right *Chi* corresponds with the *Mingmen* (Gate of Vitality); *Jing Yue Quan Shu (Complete Works of Jingyue)*, published in the Ming dynasty, suggests that the left *Chi* pulse corresponds with the bladder and large intestine, while the right *Chi* pulse corresponds with the Triple Energiser, *Mingmen*, and small intestine. Based on modern studies, the left *Chi* pulse corresponds with the kidney *Yin* and the right *Chi* pulse corresponds with the kidney *Qi* and *Mingmen* [3]. We therefore selected LBP as the condition for clarifying the relationship of the kidneys and *Chi* pulse positions, and for evaluating the effectiveness of the selected acupoints in acupuncture interventions. LBP is a common condition, ranking second amongst all musculoskeletal and connective tissue disorders affecting individuals presenting to Taiwanese TCM outpatient clinics in 2018 [15]. The relationship of the lower back and kidney is explained in the *Suwen* text in Chapter 17 of *Huang Di Nei Jing*, which states that the lower back is the “house” of the kidneys [14].

Our preliminary hypotheses were therefore the following: (1) acupuncture significantly influences the *Chi* pulse in participants with LBP, so it is an effective indicator for assessing outcomes of acupuncture intervention and (2) reduced pain intensity and improved physical movement are reflected in the FPS-R and FTF tests after acupuncture intervention.

### 2.5. Outcomes

Baseline assessments included vital signs (systolic blood pressure (SBP), diastolic blood pressure (DBP), heart rate, and body temperature), the Health Status Questionnaire, the ODI, and the CCMQ, as described in the study protocol.

The primary outcome measure was the effect of acupuncture on radial pulse patterns in individuals with LBP. The secondary outcome measures determined the effectiveness of the acupuncture treatments using the selected acupoints.

**Figure 2:**
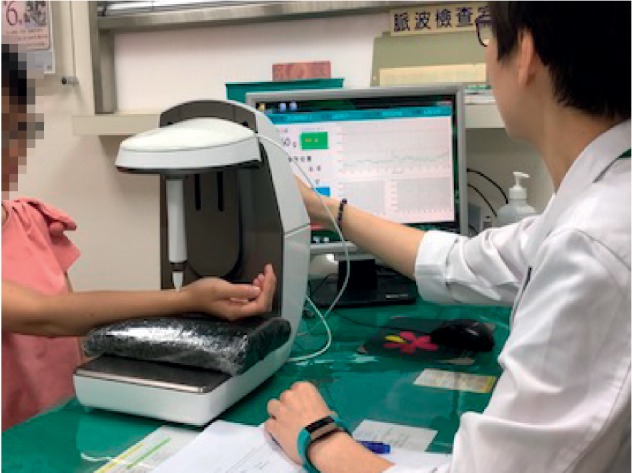
Pulse assessment of a study participant. The high-precision pressure sensor was positioned on each marked position of the *Cun*, *Guan*, and *Chi* pulses of the wrist. The biological signal of the radial pulse was then digitised to provide graphical analysis. The best spectrogram, which displayed the greatest amplitude, was then recorded.

The primary outcome measure in this study was the effect of high-frequency SE (SE_13–50 Hz_) on the radial pulse, which was assessed by the pulse sphygmograph at specific intervals during the procedure.  Figure 2 illustrates the assessment of a single study participant using the pulse sphygmograph (Pen Pulse Analysis System Model PPAS-96, Asia Plus Biotech Co., Taiwan). We selected SE_13–50 Hz_ as our primary outcome measure based on the study by Wei et al., which reported large variations in SE above 10 Hz in individuals with various diseases or mental stress [16]. We therefore anticipated significant changes would be found in SE values exceeding 10 Hz.

The secondary outcome measures used the FTF and FPS-R tests to evaluate the efficacy of the acupuncture treatments. The FTF test evaluates flexibility in the flexion ranges of motion, while the FPS-R test evaluates pain intensity. Both tests were administered during the pre- and postintervention assessments.

In this study, vital sign assessment and pulse assessment were performed during each of the baseline (*A*0), preintervention (*A*1), and postintervention (*A*2) phases, respectively. The baseline assessments were used as a control to compare the stability of these parameters at the preintervention stage. Each participant was assigned an identification code to protect the privacy of their personal details. Raw data were consolidated in Microsoft Excel 2016 and pivoted to analyse the results.

### 2.6. Design and Mechanism of the Pulse Sphygmograph

**Figure 3:**
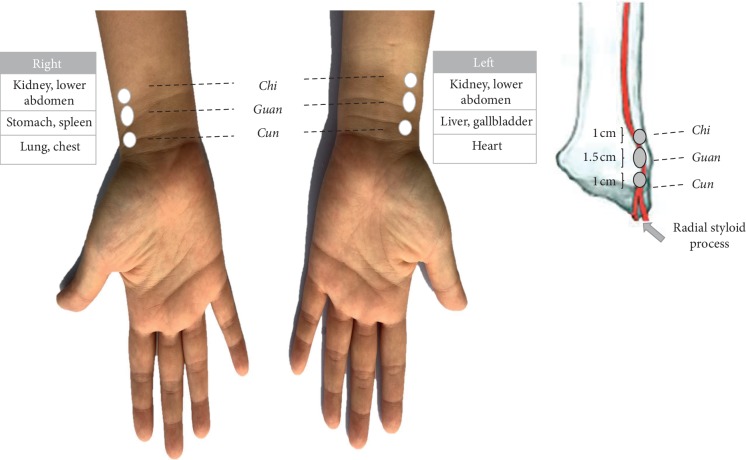
Locations of the *Cun*, *Guan*, and *Chi* pulse positions.

The pulse sphygmograph (PPAS-96) is a noninvasive diagnostic device consisting of a detachable, high-precision pulse detection sensor pen with a stable *Y*-axial movable framework. It is connected to a pulse signal analyser containing a filter, an amplifier, and a signal-recording card. The device has a sampling rate of 3,000 Hz and frequency response of 0.1–50 Hz. The input voltage uses USB_DC5V. The physiological signals of the radial pulse are digitalised and processed through the fast Fourier transform. The digital output presented in the software application includes a real-time display of the pulse spectrogram and time- and frequency-domain analyses. PPAS-96 is the enhanced model of the Huang-T1 Pulse Sphygmograph [1]. Huang-T1 Pulse Sphygmograph has been used in studies that have investigated the effects of acupuncture in patients with dyspepsia [12]; the influence of radial pulse and heart rate variability (HRV) in heat- and cold-stressed humans [17]; and a comparison of the radial pulse and HRV in normotensive and hypertensive subjects [18]. During this study assessment, we located the *Cun*, *Guan*, and *Chi* pulse positions on the left and right wrists of each study participant. The *Guan* pulse position, where the radial pulse is palpated, is located at the prominence distal to the radial styloid process (see Figure 3). The *Cun* pulse position is located at the distal aspect and the *Chi* pulse position is located at the proximal aspect of the *Guan* pulse position. An anatomical study in 1994 reported that the length of the *Guan* pulse position is approximately 1.5 cm and that of the *Cun* and *Chi* pulse positions is approximately 1 cm each [3]. In our study, we marked these pulse positions during the baseline assessment so that the same positions were subsequently assessed during the pre- and postintervention study visits.

**Figure 4:**
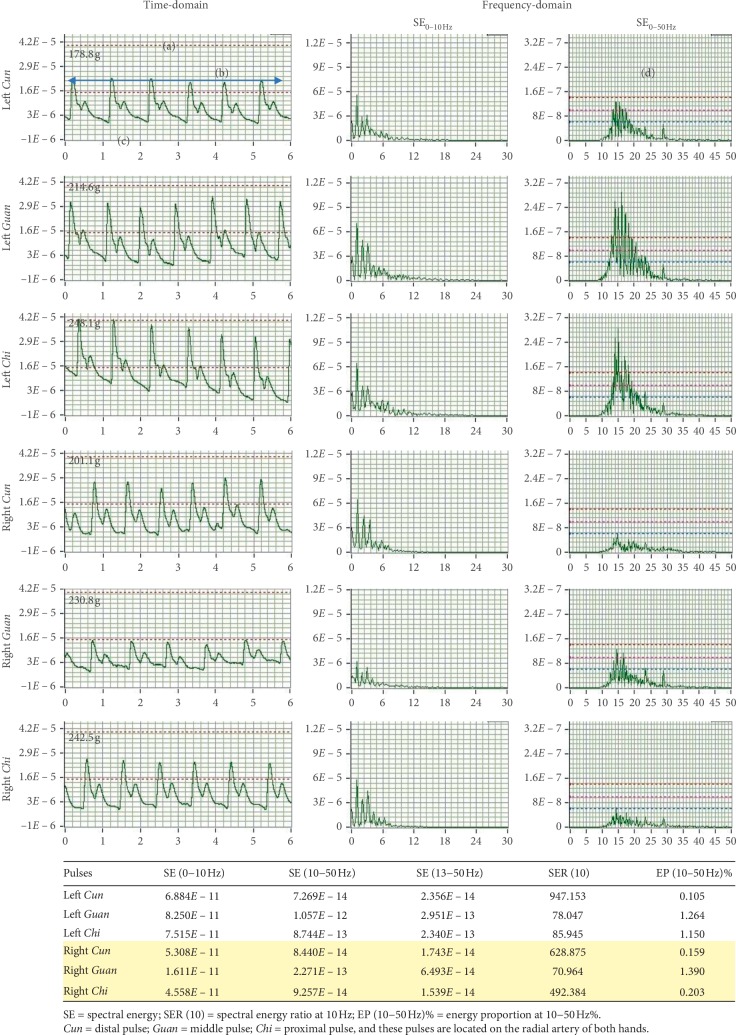
A typical report of a study participant generated from the pulse sphygmograph providing the time- and frequency-domain analyses of the six pulse positions. Each pulse profile is characterised by the following: (a) depth indicates light (0−60 g), moderate (60−120 g), and heavy (>120 g) pressure on the radial artery; (b) rate indicates the pulse rate, calculated by multiplying the number of waves by 10; (c) waveform provides information on the status of *Qi* and blood. Twenty-eight common pulse waveforms exist, as described in Introduction; and (d) density provides information on the status of heat, cold, dampness, and *Qi* stagnation in the body, while intensity indicates the amplitude of these causative factors.

Upon placing the pressure sensor pen on each pulse position, the real-time representation of the time-domain and frequency-domain analyses is displayed in the monitor. The best spectrogram with the greatest amplitude of each pulse position is sequentially recorded. A report is then generated immediately from the software application after assessment of the six pulse positions is completed (see Figure 4). In this study, we assessed the pulse positions in the same order of right *Cun*, right *Guan*, right *Chi*, left *Cun*, left *Guan*, and left *Chi* during the baseline, preintervention, and postintervention phases. There was no time lag between the measurement of each pulse position.

### 2.7. Sample Size

Shin et al. [19] and Kim et al. [20] estimated a sample size of 25 participants in their single-arm study based on a 5% type 1 error, 80% power, and 5% dropout rates, derived from the mean change and standard deviation in SE_13–50 Hz_ during the pre- and postintervention phases observed in the study by Huang et al. [12]. Huang et al. used the Huang-T1 Pulse Sphygmograph to compare the effects of acupuncture between healthy subjects and patients with dyspepsia [12]. Each study group contained 30 participants, and the reported *p* value for the difference in SE_13–50 Hz_ between the pre- and postinterventions was 0.0029; the mean difference was −3.38.

As a reference, we performed an interim analysis at the 25th trial, to examine the significant difference (*p* values) of the mean for the primary outcome. Recruitment into the study was halted after obtaining significant differences (*p* < 0.05) in SE_13–50 Hz_ and FTF and FPS-R results at the end of the approved trial period (March 2019).

### 2.8. Assignment Method and Blinding

As all participants received the same pre- and postinterventions, this study did not need to incorporate randomised assignment and blinding procedures.

### 2.9. Unit of Analysis and Statistical Methods

Analyses were performed at a group level. Baseline characteristics (age, gender, height, body weight, and vital signs), FTF and FPS-R test results, and pulse assessments were compared between *A*0 (30 min before acupuncture), *A*1 (10 min before acupuncture), and *A*2 (10 min after acupuncture). The significance of changes in the SE_13–50 Hz_ and FTF and FPS-R tests induced by the acupuncture intervention was determined using paired *t*-tests. Wilcoxon signed-rank test analysis was performed (this nonparametric statistical test is used to assess differences from matched-pair designs or repeated measures to determine whether two dependent samples selected from the populations have the same distribution). The two-sided significance level <0.05 was used. Results were analysed using SAS statistical software (Version 9.4; SAS Institute, Cary, NC, USA) and are presented as the means, standard deviations, 95% CIs, and *p* values.

### 2.10. Ethical Approval and Consent to Participate

CMUH REC, Taichung, Taiwan, approved the study under protocol nos. CMUH107-REC2-022 and CMUH107-REC2-022 (AR-1). This study was registered at www.clinicaltrials.gov (NCT03501771) on 17 April 2018. Before commencing the study, we obtained written informed consent from each participant.

## 3. Results

Forty-five individuals were enrolled into the study, and three were subsequently excluded, as they were not able to undergo pulse assessments at the principal investigator's discretion. For analysis, we excluded the data of one individual above 75 years, to reduce the error of experimental results. All study results are therefore for 41 participants. None of the enrolled study participants discontinued the study because of compliance issues or adverse events. No unintended effects or adverse events were reported in any of the assessments.

### 3.1. Baseline Characteristics

The baseline characteristics of the study population with LBP are described in Table 1. There were 29 female and 12 male participants with an average overall age of 41.63 ± 16.01 years: 48.8% were young adults (20−44 years), 39% were middle-aged (45−64 years), and 12.2% were elderly (65−75 years). LBP was reportedly felt as fixed, distended, or sharp pain by 43.9% of the study cohort; 56.1% experienced dull, aching pain (the intensity of aching was more than the intensity of sharp pain). The majority of study participants (58.5%) had minimal disabilities. Based on the CCMQ self-survey, 56.1% of the participants had balanced constitutions. The correlation between various body constitutions and different types of LBP was not analysed because of the low sample size for each type of nonbalanced constitution.

### 3.2. Vital Signs

Table 2 compares the vital signs before and after the intervention. There was no significant difference in body temperature before and after the intervention. However, there were a significant increase in DBP and an extremely significant decrease (*p* < 0.0001) in the pulse rate after the intervention.

### 3.3. Primary Outcome Measure

**Figure 5:**
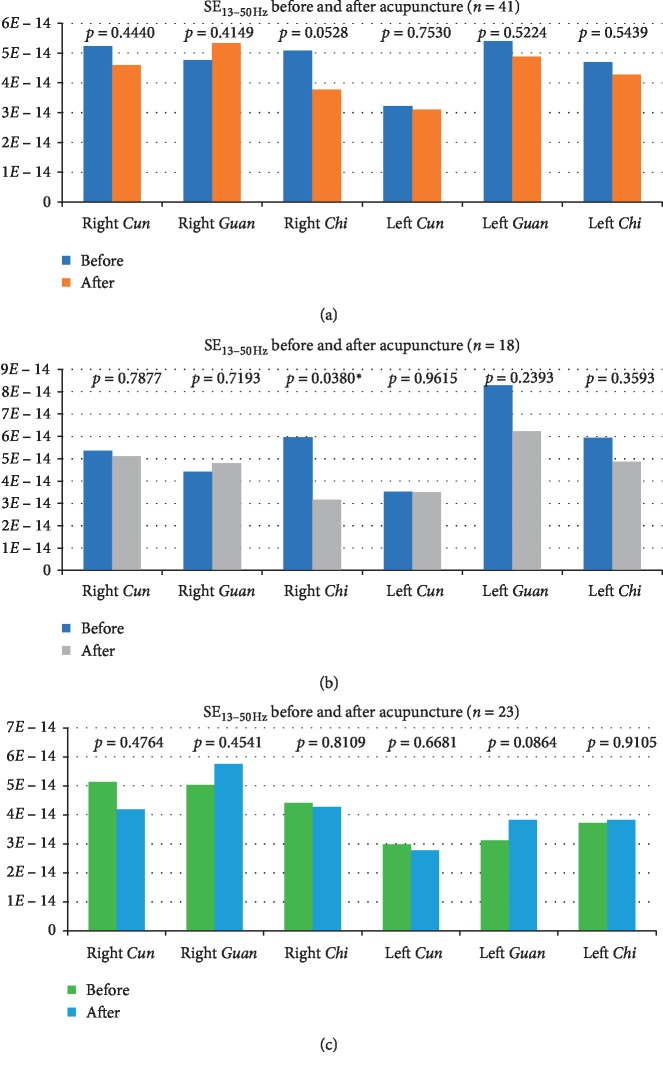
Graphical presentation of the mean SE_13–50 Hz_ determined from the pulse positions before and after acupuncture in the overall group (*n*=41) (a), Group 1 (*n*=18) (b), and Group 2 (*n*=23) (c). ^*∗*^*p* < 0.05.

The radial pulse SE_13–50 Hz_ was determined at the *Cun*, *Guan*, and *Chi* pulses before and after the intervention.  Table 3 presents the results of these six pulse positions in the overall study population. Group 1 represents study participants with fixed, distended, or sharp LBP (Table 4), and Group 2 represents those with dull, aching LBP (Table 5).  Figure 5 presents the graphical data.

The results of the radial pulse diagnoses showed that, in general, acupuncture at Shenshu (BL23), Dachangshu (BL25), and Weizhong (BL40) reduced the high-frequency SE at the right *Chi* pulse after the treatment (*p*=0.0528). The reduction was significant in Group 1 participants with fixed, distended, or sharp pain (*p*=0.0380), but not among those in Group 2 with the dull, aching pain (*p*=0.8109). In contrast, Group 2 displayed an increasing trend close to significance in high-frequency SE at the left *Guan* pulse after acupuncture treatment (*p*=0.0864).

Nonparametric analysis revealed no significant differences in SE_13–50 Hz_ during the pre- and postinterventions, except for the right *Chi* of Group 1 participants.  Table 6 provides the results of the nonparametric analysis of SE_13–50 Hz_ values for Group 1 before and after acupuncture (*A*1 vs. *A*2).

**Figure 6:**
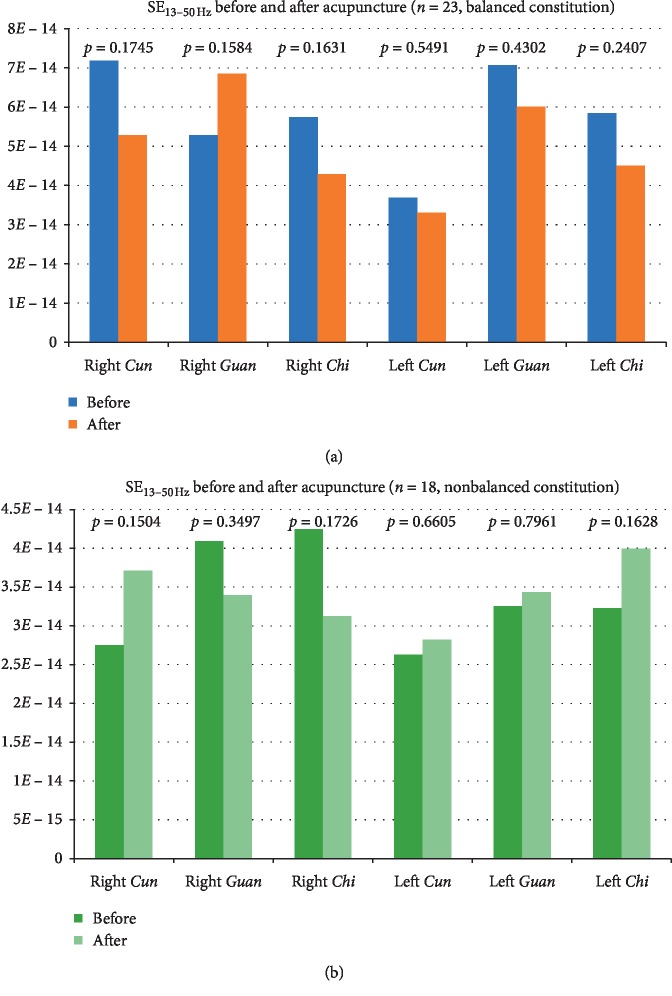
Comparison of mean SE_13–50 Hz_ values determined from the pulse positions before and after acupuncture between study participants with balanced constitutions (a) and those with nonbalanced constitutions (b).

The effects of acupuncture on the radial pulse SE_13–50 Hz_ were compared between the balanced and nonbalanced constitutions. Changes in SE_13–50 Hz_ in study participants with a balanced constitution were similar to those in Group 1 participants, although the decreases in right and left *Chi* were not significant (Figure 6). In contrast, those with nonbalanced constitutions had a nonsignificant decrease in SE_13–50 Hz_ in the right *Guan* and *Chi* and increases in the other pulse positions.

### 3.4. Secondary Outcome Measures

Table 7 provides the results of the FTF and FPS-R tests. We observed that both Group 1 and Group 2 study participants had a greater lumbar range of motion, since the distance between the floor and the finger was significantly shorter after the acupuncture session. Group 2 had significantly better lumbar range of motion than Group 1 (*p* < 0.001). FPS-R test results showed that both groups experienced a significant reduction in pain after the acupuncture procedure (*p* < 0.001), although the pain rating was significantly better for Group 2 versus Group 1.

## 4. Discussion

A pulse diagnosis provides information on the physiological and pathological states of the visceral organs and bowels [5]. This tactile method of determining essential information for diagnoses and treatments has been progressively transformed and digitised through the invention of various scientific tools over the last few decades [21]. A few studies have reported the effects of acupuncture intervention on the radial pulse in healthy subjects [20, 22–26], patients with dyspepsia [12], and those with cervical spondylosis [27]. To date, no study has investigated the effect of acupuncture on the radial pulse patterns in individuals with LBP.

### 4.1. Demographic Characteristics

**Figure 7:**
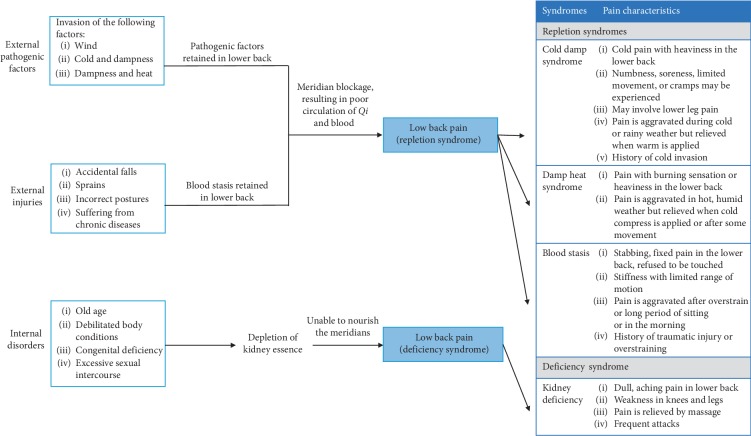
Diagrammatic presentation of aetiology and syndrome relationships of LBP. Each syndrome is characterised by the pain characteristics, associated symptoms (e.g., cold extremities in the Cold damp syndrome), and tongue and pulse characteristics.

Our study contained a higher proportion of younger adults (aged between 20 and 44 years; 48.8%) than other age groups, reflecting findings from another study demonstrating that LBP is especially prevalent among younger adults [28]. Approximately 70% of our study population was female. One study has reported that LBP is more prevalent among females than males, predominantly in occupational groups including industrial workers, white-collar workers, physiotherapists, and hospital staff [29]. As described in our published study protocol, four types of LBP exist, based on the TCM syndrome-aetiology differentiation (see Figure 7) [1].

Each LBP syndrome is identified by characteristic pain sensations and associated symptoms. For instance, a patient with Cold damp syndrome experiences cold extremities in addition to cold pain and heaviness in the lower back; numbness, soreness with limited movement, or cramps may also be experienced; the pain is worse during cold or rainy weather but relieved when warmth is applied; often experiences poor appetite and abdominal distention; has thick white-coating on the tongue; a deep and taut pulse or deep and slow pulse may be felt [30]. Although we had designed our study to recruit the four types of LBP patients, we found that only two types of pain were common in these study participants, i.e., fixed, distended, or sharp pain and dull, aching pain. Thus, to facilitate our statistical analysis, we categorised the data into two sets according to the type of pain described by the study participants, that is, Group 1 (LBP with fixed, distended, or sharp pain) and Group 2 (LBP with dull, aching pain).

About 60% of the Group 1 participants were in the acute stage (<4 weeks), while 60% of the Group 2 participants were in the chronic stage (>12 weeks). Fixed, distended, or sharp pain was experienced by study participants in the acute stage. In the TCM perspective, this was due to the obstruction of the meridians by blood stasis [31]. In contrast, dull, aching pain experienced in the chronic stage was related to kidney deficiency that resulted in poor nourishment of the meridians [32].

Over half of the study participants had a balanced body constitution based on the CCMQ survey. The CCMQ has been published by the China Association of Chinese Medicine since 2008 and is used to determine the body constitution type based on TCM concepts [33, 34]. This questionnaire contains 60 questions that categorise the body constitution into nine types, namely, Normality, *Qi*-deficiency, Yin-deficiency, Yang-deficiency, Phlegm-dampness, Damp-heat, Blood stasis, *Qi*-depressed, and Inherited special constitutions. A pilot test was carried out in a Beijing population, and its construct validity and reliability were proven by 2,500 subjects [35]. Our study sample size was not large enough to analyse correlations between types of LBP based on the nine types of body constitutions. Nevertheless, we found that the changes in SE_13–50 Hz_ after acupuncture on the radial pulse in study participants with balanced constitution were similar to the changes in SE_13–50 Hz_ in Group 1 participants. Conversely, changes in SE_13–50 Hz_ in the right *Guan* and *Chi* were nonsignificantly decreased after acupuncture in participants with nonbalanced constitutions. These results are inconclusive because of a lack of significance. It may be worthwhile conducting future clinical studies with larger sample sizes.

### 4.2. Vital Signs

We observed an insignificant elevation in SBP but a significant elevation in DBP within the normal range after bilateral acupuncture intervention at BL23, BL25, and BL40. This phenomenon has not been previously reported by any studies. Flachskampf et al. reported that acupuncture significantly reduced SBP and DBP in hypertensive patients in a randomised controlled trial [36]. In dogs with low blood pressure, electroacupuncture at Zusanli (ST36) or Neiguan (PC6) significantly restored blood pressure to normal levels [37]. To explain this phenomenon using the TCM meridian theory, the *Suwen* text in Chapter 24 of *Huang Di Nei Jing* describes how “the Tai-Yang meridians often have more blood and less *Qi*” [14]. We therefore speculate that when we stimulated the acupoints on the Bladder meridian of the Foot Tai-Yang, blood circulation was improved. Moreover, Wang et al. used a blood perfusion imaging technique to conclude that acupuncture at BL40 can increase skin blood perfusion in the lower back [38]. Further investigations are warranted to obtain more conclusive results on the comparative effects of specific acupoints on blood pressure.

Our findings also show that the pulse rate was significantly reduced within the 20-min interval before the intervention. There was a marked significant decrease after the intervention. Streitberger et al. have pointed out that the decrease in the heart rate after an acupuncture procedure could be due to delayed vagal activation [39].

### 4.3. Primary Outcome Measure: Radial Pulse Diagnosis

The results of the radial pulse SE_13–50 Hz_ analysis suggest that our primary hypothesis holds true, that is, “acupuncture can significantly influence the *Chi* pulse in participants with LBP.” The SE_13–50 Hz_ of the right *Chi* pulse was significantly reduced after the acupuncture treatment at the bilateral BL23, BL25, and BL40 (*p*=0.0528). However, we observed that Group 1 participants had a more significant reduction (*p*=0.0380) compared to those in Group 2 (*p*=0.6903). In contrast, Group 2 participants had a marked trend of increasing SE at the left *Guan* pulse after the treatment (*p*=0.0864).

For the subgroup analyses, the only significant difference in SE_13–50 Hz_ was found in the right *Chi* of Group 1 study participants between the pre- and postinterventions and was probably a result of the small sample size. In further analysis using the Wilcoxon signed-rank test, the nonparametric results showed less significant difference in SE_13–50 Hz_ of right *Chi* for the overall population (*n*=41) before and after acupuncture. Subgroup analysis of Group 1 maintained robust significance in the comparison of right *Chi*. The nonparametric analysis showed the same trend and consistent result as the parametric analysis. Thus, these results provided valuable information and suggested that the right *Chi* pulse can effectively assess the effect of acupuncture intervention in LBP patients with fixed, distended, or sharp pain, while the left *Guan* pulse is potentially useful to assess those with dull, aching pain.

We speculated that higher density and intensity of SE were generally found at the right *Chi* pulse of Group 1 study participants. The high SE corresponded to the increased vasomotion of the peripheral blood vessels. This coincides with Huang et al.'s study, which reported that heat stress subjects had an elevated SE that resulted from an increase in peripheral vascular dilatation and vasomotion [17]. The increased vasomotion was a result of the elevated sympathetic nervous activity stimulated by pain [40]. However, the effects of acupuncture on sympathetic nerve activity are controversial. Middlekauff et al. concluded that acupuncture inhibited sympathetic nerve activity during mental stress in patients with heart failure [41], whereas Kim et al. reported that acupuncture at ST36 increased sympathetic nerve activity in healthy participants [20]. In this study, we speculate that bilateral acupuncture at BL23, BL25, and BL40 attenuated sympathetic nerve activity, reducing the contraction force of local muscles and vasomotion. Thus, pain was relieved, and a high-frequency SE at the right *Chi* pulse was decreased.

*Huang Di Nei Jing* states that “both *Chi* pulse positions correspond to the kidney and the abdomen” and that “the lumbus represents the house of the kidneys” [3, 14]. Interestingly, we found that bilateral acupuncture at BL23, BL25, and BL40 acupoints for treatment of LBP influences high-frequency SE more significantly at the right *Chi* pulse rather than both *Chi* pulses. To explain this phenomenon, we revisited the ancient literature. *Nan Jing*: *The Classic of Difficult Issues* states that the *Chi* pulses correspond to the bladder [3]. In clinical practice, the right *Chi* pulse is more closely associated with the bladder, although some physicians and philosophers have suggested that the left *Chi* pulse corresponds to the bladder [3]. The *Ling Shu* text in Chapter 10 states that the Bladder meridian of the Foot *Tai-Yang* is affiliated with the bladder and connects to the kidneys. In addition, *Shang Han Lun (Treatise on Cold Pathogenic Diseases)* states that the *Tai-Yang* meridians dominate the body's exterior. We speculated that fixed, distended, or sharp LBP results from kidney *Qi* repletion caused by the invasion of pathogenic factors on the body's exterior, mainly influencing pulse density and intensity and causing a large variation in high-frequency SE [16]. Thus, higher density and intensity of SE were found at the right *Chi* pulse before treatment. Acupuncture at BL23, BL25, and BL40 of the Bladder meridian resulted in decreased density and intensity of SE, reflecting directly on the right *Chi* pulse. Future studies could investigate the effects of acupoints from other meridians associated with the treatment of LBP on the left and right *Chi* pulses, to prove our speculation. The *Suwen* text in Chapter 39 of *Huang Di Nei Jing* describes how sudden pain occurs when the *Qi* channel is obstructed [14]. Conversely, a dull, aching pain is caused by *Qi* and blood insufficiency [42]. Our findings showed an increased high-frequency SE at the left *Guan* pulse of Group 2 participants after their acupuncture treatment. We speculate that these individuals have a circulatory insufficiency. A significant increase in blood flow during these acupuncture sessions resulted in a higher intensity of the SE in the left *Guan*, as it corresponds to the liver's blood supply and nervous system in TCM. Takayama et al. reported a significant increase in blood flow through the superior mesenteric artery after acupuncture stimulation at ST36 [43]. Our study also found a significant elevation in DBP within the normal range after the acupuncture. A future study may be warranted to investigate the effect of blood flow in the hepatic artery during stimulation of BL23, BL25, and BL40, to confirm the phenomenon observed in our study.

### 4.4. Secondary Outcome Measures: Pain Intensity and Physical Flexibility

The results from our assessments of lumbar ranges of motion and pain intensity suggest that our secondary hypothesis holds true, that is, “reduced pain intensity and better physical movement can be reflected in the FTF and FPS-R tests after the acupuncture intervention.” Generally, there were a significant reduction in pain intensity and improvement in the lumbar range of motion after acupuncture stimulation. This observation was also reported by Furlan et al., who found that acupuncture treatments provided a more effective functional improvement and pain relief than sham treatments or no treatment [44]. We observed a marked significant difference (*p* < 0.001) in Group 2 participants than in Group 1 participants, which suggests that BL23, BL25, and BL40 are effective for the treatment of dull, aching LBP. Studies have shown that the nociceptive pathways, including descending serotonergic and noradrenergic pathways, may be involved in the immediate analgesic effect of acupuncture on various types of pain [45]. More studies are needed to compare the analgesic effects of acupuncture on different types of pain.

### 4.5. Study Limitations and Solutions

Although quasi-experimental research is more feasible than true experimental research as it requires less time and has fewer logistical constraints, there were several limitations in our study. Because of the small sample size of this pilot study, the lack of random assignments in the quasi-experimental design may have presented a confounding bias that may limit the generalizability of the findings to a larger population. We therefore identified the threats to the validity of our findings in our experimental design to reduce the impact. For instance, we used the baseline assessments of the radial pulse and vital signs as a control to compare the stability of these parameters before the acupuncture intervention; the same acupuncturist applied a consistent acupuncture technique at the same acupoints in the treatment, and the acupuncturist was not informed of the type of LBP experienced by the study participants, in an attempt to avoid bias. Future studies with larger sample sizes and randomisation procedures are warranted to provide more conclusive results. We did not anticipate the outcome demonstrating how acupuncture differentially affects high-frequency SE in different types of LBP. Future research is warranted to compare treatment outcomes between healthy subjects and those with LBP. Another limitation was that no other pulse diagnostic device measures the SE_13–50 Hz_ besides the approved PPAS-96 and Huang-T1 Pulse Sphygmograph in Taiwan. Therefore, we were not able to compare the outcomes with other devices. However, Huang-T1 Pulse Sphygmograph has been used in a few clinical studies [12, 17, 18], as well as in the Taiwanese hospitals and clinics; we therefore used this device in our preliminary study to assess the feasibility of the protocol design before proceeding with PPAS-96 Pulse Sphygmograph [1]. It will be beneficial to conduct future studies to compare and verify the SE measurements with other devices that will be available.

## 5. Conclusion

Our study provides novel information about the effects of acupuncture on the radial pulse SE in individuals with different types of LBP. Our findings suggest that the right *Chi* pulse is an effective indicator for assessing the effects of acupuncture intervention in individuals with fixed, distended, or sharp pain, whereas the left *Guan* pulse is a potentially useful diagnostic technique to determine acupuncture's effects in those with dull, aching pain. The acupoints BL23, BL25, and BL40 provide effective treatment for LBP. Individuals with dull, aching pain had a significant improvement in their lumbar ranges of motion, and they showed a noteworthy decrease in pain ratings after receiving acupuncture treatment.

## Figures and Tables

**Table 1:** Baseline characteristics of the study participants with lower back pain.

Baseline characteristics	Number	Percentage
Total number of patients	**41**	

Gender		
Female	**29**	70.7
Male	**12**	29.3

Age (years)		
20–44	**20**	48.8
45–64	**16**	39.0
65–75	**5**	12.2

Types of LBP		
Fixed, distended, or sharp pain (Group 1)	**18**	43.9
Acute (<4 weeks)	11	
Subacute (4–12 weeks)	2	
Chronic (>12 weeks)	5	
Dull, aching pain (Group 2)	**23**	56.1
Acute (<4 weeks)	8	
Subacute (4–12 weeks)	1	
Chronic (>12 weeks)	14	

ODI		
Minimal disability	**24**	58.5
Moderate disability	**13**	31.7
Severe disability	**4**	9.8

CCMQ		
Balanced constitution	**23**	56.1
Nonbalanced constitution (*Qi-*deficient, Yang-deficient, Yin-deficient, Phlegm-dampness, Damp-heat, Stagnant blood, Stagnant *Qi*, and Inherited Special Constitutions)	**18**	43.9

ODI: Oswestry Disability Index; CCMQ: Constitution in Chinese Medicine Questionnaire.

**Table 2:** Comparison of the vital signs at baseline and before and after the intervention.

Parameter	Baseline (*A*0)	Preintervention (*A*1)	Postintervention (*A*2)	*p* value (*A*0 vs. *A*1)	*p* value (*A*1 vs. *A*2)	95% CI (*A*0 vs. *A*1)	95% CI (*A*1 vs. *A*2)
Body temperature (°C)	36.46 ± 0.39	36.53 ± 0.42	36.45 ± 0.39	0.0982	0.0704	−0.1498, 0.0132	−0.0068, 0.1629
SBP (mmHg)	116.17 ± 17.56	114.46 ± 17.76	115.22 ± 16.76	0.0593	0.5270	−0.0702, 3.4848	−3.1504, 1.6382
DBP (mmHg)	74.76 ± 12.49	73.02 ± 12.24	76.44 ± 12.54	**0.0315** ^*∗*^	**0.0039** ^*∗*^	0.1614, 3.3020	−5.6698, −1.1594
Pulse rate (beats/min)	71.87 ± 11.14	69.39 ± 11.04	65.26 ± 9.53	**0.0001** ^*∗∗*^	**7.59*E* − 08** ^*∗∗*^	1.4612, 4.0022	3.1063, 5.8693

Values are presented as mean ± SD. ^*∗*^*p* < 0.05; ^*∗∗*^*p* < 0.001. *A*0 = assessed 30 min before acupuncture; *A*1 = assessed 10 min before acupuncture; *A*2 = assessed 10 min after acupuncture; DBP: diastolic blood pressure; SBP: systolic blood pressure; SD = standard deviation. 95% CI is presented as a range from the lower limit to the upper limit.

**Table 3:** SE_13–50 Hz_ of the six pulse positions (all, *n*=41).

Pulse position		Baseline (*A*0)	Preintervention (*A*1)	Postintervention (*A*2)	*p* value (*A*0 vs. *A*1)	*p* value (*A*1 vs. *A*2)	95% CI (*A*0 vs. *A*1)	95% CI (*A*1 vs. *A*2)
Right *Cun*	Mean	4.093*E* − 14	5.235*E* − 14	4.590*E* − 14	0.1696	0.4440	1.80*E* − 14, 1.40*E* − 13	1.09*E* − 13, 7.77*E* − 14
	SD	5.386*E* − 14	6.165*E* − 14	3.811*E* − 14				

Right *Guan*	Mean	4.972*E* − 14	4.758*E* − 14	5.333*E* − 14	0.7599	0.4149	−1.01*E* − 13, 1.08*E* − 13	−7.85*E* − 14, 1.02*E* − 13
	SD	4.444*E* − 14	3.004*E* − 14	4.710*E* − 14				

Right *Chi*	Mean	5.077*E* − 14	5.086*E* − 14	3.779*E* − 14	0.9794	**0.0528**	−2.90*E* − 14, 8.21*E* − 14	−1.47*E* − 13, 1.85*E* − 14
	SD	5.560*E* − 14	4.643*E* − 14	2.773*E* − 14				

Left *Cun*	Mean	3.588*E* − 14	3.219*E* − 14	3.095*E* − 14	0.3809	0.7530	−7.20*E* − 14, 4.80*E* − 14	−4.97*E* − 14, 4.87*E* − 14
	SD	3.086*E* − 14	2.271*E* − 14	2.047*E* − 14				

Left *Guan*	Mean	4.543*E* − 14	5.394*E* − 14	4.880*E* − 14	0.3432	0.5224	−6.08*E* − 14, 6.99*E* − 14	−3.47*E* − 14, 7.22*E* − 14
	SD	4.923*E* − 14	8.254*E* − 14	4.957*E* − 14				

Left *Chi*	Mean	4.129*E* − 14	4.696*E* − 14	4.279*E* − 14	0.4741	0.5439	−4.90*E* − 14, 8.82*E* − 14	−4.80*E* − 14, 7.99*E* − 14
	SD	3.991*E* − 14	6.041*E* − 14	4.235*E* − 14				

^*∗*^ *p* < 0.05. *A*0 = assessed 30 min before acupuncture; *A*1 = assessed 10 min before acupuncture; *A*2 = assessed 10 min after acupuncture; SD = standard deviation. 95% CI is presented as a range from the lower limit to the upper limit.

**Table 4:** SE_13–50 Hz_ of the six pulse positions of study participants with fixed, distended, or sharp pain (Group 1, *n*=18).

Pulse position		Baseline (*A*0)	Preintervention (*A*1)	Postintervention (*A*2)	*p* value (*A*0 vs. *A*1)	*p* value (*A*1 vs. *A*2)	95% CI (*A*0 vs. *A*1)	95% CI (*A*1 vs. *A*2)
Right *Cun*	Mean	4.578*E* − 14	5.360*E* − 14	5.104*E* − 14	0.3843	0.7877	–263*E* − 16, 1.07*E* − 14	–171*E* − 16, 2.23*E* − 14
	SD	5.672*E* − 14	5.759*E* − 14	4.740*E* − 14				

Right *Guan*	Mean	5.681*E* − 14	4.407*E* − 14	4.795*E* − 14	0.3307	0.7193	–141*E* − 16, 3.96*E* − 14	–263*E* − 16; 1.85*E* − 14
	SD	5.542*E* − 14	2.905*E* − 14	4.021*E* − 14				

Right *Chi*	Mean	6.109*E* − 14	5.961*E* − 14	3.155*E* − 14	0.7264	**0.0380** ^*∗*^	–731*E* − 17, 1.03*E* − 14	1.75*E* − 15, 5.44*E* − 14
	SD	6.233*E* − 14	5.827*E* − 14	1.772*E* − 14				

Left *Cun*	Mean	3.772*E* − 14	3.531*E* − 14	3.497*E* − 14	0.6093	0.9615	–735*E* − 17, 1.22*E* − 14	–145*E* − 16, 1.52*E* − 14
	SD	3.532*E* − 14	2.432*E* − 14	2.453*E* − 14				

Left *Guan*	Mean	5.728*E* − 14	8.294*E* − 14	6.220*E* − 14	0.2036	0.2393	–666*E* − 16, 1.53*E* − 14	–151*E* − 16, 5.66*E* − 14
	SD	6.785*E* − 14	1.180*E* − 13	6.894*E* − 14				

Left *Chi*	Mean	5.339*E* − 14	5.929*E* − 14	4.857*E* − 14	0.7167	0.3593	–396*E* − 16, 2.78*E* − 14	–133*E* − 16, 3.47*E* − 14
	SD	5.309*E* − 14	8.296*E* − 14	5.285*E* − 14				

^*∗*^ *p* < 0.05. *A*0 = assessed 30 min before acupuncture; *A*1 = assessed 10 min before acupuncture; *A*2 = assessed 10 min after acupuncture; SD = standard deviation. 95% CI is presented as a range from the lower limit to the upper limit.

**Table 5:** SE_13–50 Hz_ of the six pulse positions of patients with dull, aching pain (Group 2, *n*=23).

Pulse position		Baseline (*A*0)	Preintervention (*A*1)	Postintervention (*A*2)	*p* value (*A*0 vs. *A*1)	*p* value (*A*1 vs. *A*2)	95% CI (*A*0 vs. *A*1)	95% CI (*A*1 vs. *A*2)
Right *Cun*	Mean	3.714*E* − 14	5.137*E* − 14	4.187*E* − 14	0.2852	0.4764	−2.90*E* − 14, 1.40*E* − 13	−1.77*E* − 13, 1.30*E* − 13
	SD	5.249*E* − 14	6.593*E* − 14	2.941*E* − 14				

Right *Guan*	Mean	4.417*E* − 14	5.032*E* − 14	5.754*E* − 14	0.4033	0.4541	−9.50*E* − 14, 2.18*E* − 13	−1.20*E* − 13, 1.54*E* − 13
	SD	3.382*E* − 14	3.116*E* − 14	5.236*E* − 14				

Right *Chi*	Mean	4.269*E* − 14	4.402*E* − 14	4.268*E* − 14	0.8138	0.8109	−3.16*E* − 14, 1.25*E* − 13	−1.34*E* − 13, 1.07*E* − 13
	SD	4.962*E* − 14	3.441*E* − 14	3.315*E* − 14				

Left *Cun*	Mean	3.445*E* − 14	2.975*E* − 14	2.780*E* − 14	0.4826	0.6681	−1.03*E* − 13, 4.70*E* − 14	−4.80*E* − 14, 5.12*E* − 14
	SD	2.762*E* − 14	2.160*E* − 14	1.654*E* − 14				

Left *Guan*	Mean	3.616*E* − 14	3.125*E* − 14	3.832*E* − 14	0.1119	0.0864	−1.21*E* − 13, 1.14*E* − 14	−3.95*E* − 15, 1.22*E* − 13
	SD	2.540*E* − 14	1.987*E* − 14	2.309*E* − 14				

Left *Chi*	Mean	3.182*E* − 14	3.731*E* − 14	3.826*E* − 14	0.4207	0.9105	−6.59*E* − 14, 1.08*E* − 13	−8.01*E* − 14 1.60*E* − 13
	SD	2.246*E* − 14	3.310*E* − 14	3.246*E* − 14				

*A*0 = assessed 30 min before acupuncture; *A*1 = assessed 10 min before acupuncture; *A*2 = assessed 10 min after acupuncture; SD = standard deviation. 95% CI is presented as a range from the lower limit to the upper limit.

**Table 6:** Comparison of the SE_13–50 Hz_ of Group 1 (*n*=18) before and after acupuncture.

Pulse position		Preintervention (*A*1)	Postintervention (*A*2)	*p* value (*A*1 vs. *A*2)
Right *Cun*	Mean	5.360*E* − 14	5.104*E* − 14	0.551
	SD	5.759*E* − 14	4.740*E* − 14	

Right *Guan*	Mean	4.407*E* − 14	4.795*E* − 14	1.000
	SD	2.905*E* − 14	4.021*E* − 14	

Right *Chi*	Mean	5.961*E* − 14	3.155*E* − 14	**0.043** ^*∗*^
	SD	5.827*E* − 14	1.772*E* − 14	

Left *Cun*	Mean	3.531*E* − 14	3.497*E* − 14	0.965
	SD	2.432*E* − 14	2.453*E* − 14	

Left *Guan*	Mean	8.294*E* − 14	6.220*E* − 14	0.364
	SD	1.180*E* − 13	6.894*E* − 14	

Left *Chi*	Mean	5.929*E* − 14	4.857*E* − 14	0.806
	SD	8.296*E* − 14	5.285*E* − 14	

^*∗*^ *p* < 0.05. *A*1 = assessed 10 min before acupuncture; *A*2 = assessed 10 min after acupuncture; SD = standard deviation. Values are presented as mean ± SD.

**Table 7:** Results of the secondary outcome measures, the fingertip-to-floor (FTF) test, and the Faces Pain Scale-Revised (FPS-R) test.

Parameter	Preintervention (*A*1)	Postintervention (*A*2)	*p* value (*A*1 vs. *A*2)	95% CI (*A*1 vs. *A*2)
Fingertip-to-floor (FTF) (cm)	12.59 ± 12.27	9.88 ± 10.21	**0.0001** ^*∗∗*^	1.4078, 4.0068
Group 1	16.36 ± 14.67	12.33 ± 11.76	**0.0070** ^*∗*^	1.2571, 6.7985
Group 2	9.63 ± 8.87	7.96 ± 8.28	**0.0001** ^*∗∗*^	0.7786, 2.5692

Faces Pain Scale (FPS-R)	3.56 ± 1.64	1.22 ± 1.67	**1.24*E* − 12** ^*∗∗*^	1.8754, 2.8076
Group 1	4.22 ± 1.80	1.89 ± 1.88	**2.25*E* − 05** ^*∗∗*^	1.4805, 3.1862
Group 2	3.04 ± 1.33	0.70 ± 1.29	**1.55*E* − 08** ^*∗∗*^	1.7854, 2.9103

Values are presented as mean ± SD. ^*∗*^*p* < 0.05; ^*∗∗*^*p* < 0.001. *A*1 = assessed 10 min before acupuncture; *A*2 = assessed 10 min after acupuncture; SD = standard deviation. 95% CI is presented as a range from the lower limit to the upper limit.

## Data Availability

The data used to support the findings of this study are available from the corresponding author upon request.
